# A 12-month prospective exploratory study of muscle and fat characteristics in individuals with mild-to-moderate hip osteoarthritis

**DOI:** 10.1186/s12891-019-2668-z

**Published:** 2019-06-14

**Authors:** Aderson Loureiro, Maria Constantinou, Belinda Beck, Rod S. Barrett, Laura E. Diamond

**Affiliations:** 1 School of Allied Health Sciences, Griffith University, Gold Coast, Australia; 2 Pontifical Catholic University (PUCRS), Porto Alegre, Brazil; 3 Faculty of Physical Education and Sports, University of Rio dos Sinos (UNISINOS), São Leopoldo, Brazil; 4 School of Physiotherapy, Australian Catholic University, Brisbane, Australia; 5 Gold Coast Orthopaedics Research Engineering & Education Alliance (GCORE), Griffith University, Menzies Health Institute Queensland, Gold Coast, Australia; 6 Centre of Clinical Research Excellence in Spinal Pain, Injury & Health, School of Health & Rehabilitation Sciences The University of Queensland, Brisbane, Australia

**Keywords:** Strength, Morphology, Hip joint, Lean mass, Density

## Abstract

**Background:**

Reductions in lower extremity muscle strength, size and quality and increased fat content have been reported in advanced hip osteoarthritis (OA). Whether these differences are also evident at earlier stages of the disease and the extent to which they might develop over time is unclear. The main purpose of this 12-month exploratory prospective study was to compare changes in muscle and fat characteristics in individuals with mild-to-moderate hip OA and healthy controls.

**Methods:**

Fourteen individuals with mild-to-moderate symptomatic and radiographic hip OA (*n* = 9 unilateral; *n* = 5 bilateral), and 15 healthy controls similar in age and sex without symptoms or radiographic hip OA were assessed at baseline and at 12-month follow-up. Maximal voluntary isometric strength of the hip and knee muscle groups was assessed using an isokinetic dynamometer. Lower extremity lean and fat mass were assessed using dual-energy x-ray absorptiometry, and thigh muscle and fat areas and thigh muscle density were assessed using peripheral quantitative computed tomography.

**Results:**

Knee extension (*p* = 0.01), hip extension (*p* < 0.01), hip flexion (*p* = 0.03), and hip abduction (*p* < 0.01) strength, lower extremity lean mass (*p* < 0.01), thigh muscle area (*p* = 0.03), and thigh muscle density (*p* < 0.01) were significantly lower in hip OA compared to controls. Hip extension (*p* < 0.05), hip flexion (*p* = 0.03), and hip abduction (*p* = 0.03) strength significantly declined over the follow-up period in the hip OA group.

**Conclusions:**

Pre-existing deficits in hip muscle strength in individuals with mild-to-moderate hip OA were accentuated over 12-months, though no changes in symptoms or joint structure were observed. A longer follow-up period is required to establish whether strength deficits drive clinical and structural decline in these patients. Interventions to prevent or slow declines in strength may be relevant in the management of mild-to-moderate hip OA.

**Electronic supplementary material:**

The online version of this article (10.1186/s12891-019-2668-z) contains supplementary material, which is available to authorized users.

## Background

Hip osteoarthritis (OA) is a prevalent and costly chronic musculoskeletal condition and a leading cause of disability worldwide [1]. Pain is a dominant symptom of hip OA, often leading to reduced physical function and quality of life. From a structural perspective, hip OA is characterised by loss of articular cartliage and changes to subcondral bone, but other tissues including muscle are also adverserly affected [2, 3]. Individuals with advanced hip OA have lower isometric, concentric and eccentric hip muscle strength than healthy controls [4], which likely underpins the reported reduction in physical function in these patients [5, 6]. Smaller muscle size appears to be a principle mechanism underlying hip muscle weakness in individuals with advanced hip OA [7], although lower muscle activation capacity (i.e. muscle inhibition [8]) and lower muscle quality (i.e. higher intra-muscular fat [7, 9]) may also play a role. At present, the pattern and underlying mechanism of lower limb muscle weakness in earlier stages of the disease, and the extent to which muscle weakness might develop over time is unclear. If atrophic lower limb muscle weakness is indeed a feature of earlier stages of the disease than previously reported [7], early intervention to mitigate these deficits may be warranted.

Some individuals with structural hip OA exhibit no clinical symptoms or structural progression for decades, while approximately 40% of those with painful hip OA will undergo total hip replacement surgery within two years [10, 11]. An improved understanding of the patient-specific pathogenesis of hip OA is required to explain the heterogeneous presentation of hip OA and to allow for targeted disease modifying interventions to be implemented early in the disease course. A range of factors have been implicated in the pathogenesis of hip OA which may be broadly categorised as person-level (i.e. obesity, age, sex) and joint-level (i.e. joint misalignment, joint morphology, joint mechanics) factors [12, 13]. These factors tend to affect either the biomechanical or the metabolic environment of the joint. For example, joint misalignment and/or altered joint morphology can disrupt the normal mechanical function of the hip joint [12]. An optimal amount and direction of tissue loading is needed to maintain bone and cartilage health, with either abnormal or unaccustomed loading generally considered detrimental [14, 15]. The forces generated by the hip-spanning muscles contribute substantially to the loads incurred by the hip joint [16]. Hip muscle weakness could therefore promote altered mechanical function of the joint in hip OA patients, and have important implications for disease progression.

Individuals with lower limb OA are reported to have lower lean body mass [17] and a greater proportion of fat in their muscles and bones compared to healthy controls [18, 19]. Increased fat in hip OA could adversely affect the hip joint via increased mechanical loading in combination with systemtic inflammation and localised lipotoxic effects on joint tissues [20]. Obesity [21] and higher intra-muscular fat [7] are common in individuals with advanced hip OA. To date, there are no known prospective studies indicating whether alterations to lower extremity lean and fat mass and the properties of muscles surrounding the hip joint (e.g. thigh muscle size and quality) also occur in individuals with mild-to-moderate hip OA.

The purpose of this 12-month prospective study was to identify changes in isometric muscle strength, muscle size and quality, and lower extremity lean and fat mass in individuals with mild-to-moderate hip OA relative to healthy controls without hip OA. We hypothesized that muscle strength, size and quality, and lower extremity lean mass would be lower and fat mass higher in hip OA patients relative to controls. Greater declines in muscle strength, size and quality and greater increases in fat mass and area were expected in the hip OA group over the 12-month follow-up period.

## Methods

### Participants

This study included participants from another study [22] who were available for follow-up. Fourteen volunteers (45–80 years) with symptomatic hip OA were recruited from local hospital orthopaedic waiting lists. Fifteen controls similar in age and sex were recruited through advertising and word-of-mouth. All participants were screened through self-reported measures of pain and function (modified Harris Hip Score (HHS) [23]) and radiographic examination (anterior-posterior radiographs of the pelvis and hips). Bilateral weight-bearing radiographs were acquired with participants in standing, on a custom built platform with foot map [24] and feet internally rotated by 15-degrees [25]. Bilateral supero-medial, apical and supero-lateral hip joint space width (JSW) were measured [26]. Kellgren-Lawrence (KL) grade [27] and JSW were assigned by a single blinded radiologist. Participants were included in the hip OA group if they had hip pain and/or functional limitations during activities of daily living (HHS ≤ 95; 0 = extreme hip problems, 100 = no hip problems) and a KL grade for their affected hip(s) of 2 or 3 and/or joint space width ≤ 3 mm; unilateral hip OA participants had KL scores of 0 or 1 for their contralateral hip. Participants in the healthy control group did not have hip pain or functional limitations during activities of daily living (HHS > 95) and had KL grades ≤1 and joint space widths > 3 mm for both hips. Exclusion criteria were: (i) previous lower limb or back fracture or surgery; (ii) history of trauma to the hip joint or pelvis region; (iii) other forms of arthritis, diabetes, cardiac or circulatory conditions; and (iv) use of corticosteroid medication. All participants could walk without physical assistance/devices.

This prospective exploratory study used a convenience sample of participants enrolled in other studies [22, 28]. Participants provided written informed consent. Ethics approval was obtained from the Institutional Human Research Ethics Committee.

### Procedures

Participants underwent strength testing, dual-energy x-ray absorptiometry (DXA), and peripheral quantitative computed tomography (pQCT) during a single session at both baseline and 12-month follow-up. All measures were acquired for the affected (unilateral OA), most symptomatic (bilateral OA), or randomly assigned test limb (control). This study conformed to the STROBE statement for reporting observational studies [29]. The primary outcome measure was change in isometric muscle strength in individuals with mild-to-moderate hip OA compared to controls. Secondary outcome measures of change in muscle size and quality, and lower extremity lean and fat mass were also explored.

Maximal voluntary isometric strength of the hip and knee flexors and extensors, and the hip abductors and adductors were measured with an isokinetic dynamometer (Biodex System 4, Biodex Medical Systems, USA) (Additional file 1: Figure S1). Complete details of these strength measures are reported elsewhere [22]. Test-retest repeatability of the muscle strength protocol used in the present study was previously evaluated in our lab in a cohort of healthy, community dwelling older adults (*n* = 60) and yielded intra-class correlation coefficients in the range 0.91 to 0.94 and minimum detectable changes in the range 11–20 Nm. Isometric strength was calculated as the peak torque normalized to body mass (Nm/kg).

Lower extremity lean and fat mass (g/kg) were assessed via a whole body DXA (Norland XR800, Cooper Surgical, USA). Lean and fat mass scans were acquired with the participant supine, palms down and legs slightly apart. The region of interest included the femoral neck and lower limb but excluded the pelvis. Data processing was conducted using Illuminatus software (V.4.2.4) with standardised positioning of the femoral neck box.

**Fig. 1:**
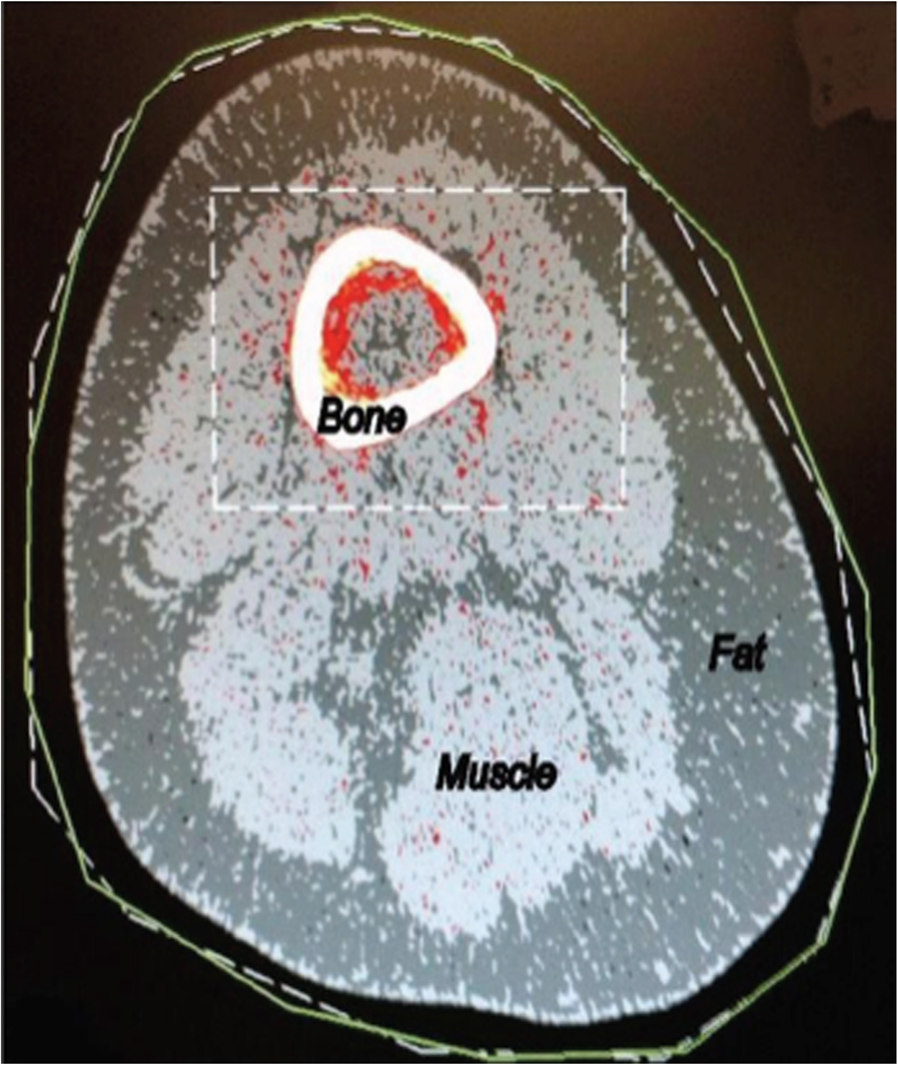
Representative peripheral quantitative computed tomography scan of the thigh for a participant with hip osteoarthritis depicting muscle and fat area

Muscle and fat cross-sectional areas (cm^2^/kg) and muscle density (mg/cm^3^) were assessed using pQCT (Stratec model XCT 3000, Medizintechnic, Pforzheim, Germany). Calibration of pQCT was achieved by standard daily quality assurance scans of a reference phantom [30]. Participants were seated with the hip in 90 degrees of flexion and the knee fully extended. The test limb was secured with a Velcro wrap to prevent movement during the scan. The base of the notch of the intercondylar fossa on scout scan was the distal reference point for scan plane standardization. The scan slice was acquired proximal to the reference point by a distance of 25% of the femoral length using an in-plane voxel size of 300 μm and a slice thickness of 2.3 mm (Fig. 1). Test-retest repeatability of muscle cross-sectional area and muscle density measures using pQCT was previously evaluated in our lab (*n* = 34) and yielded coefficients of variation of less than 1%, indicating high levels of repeatability.

### Statistical analysis

Independent t-tests or Pearson’s chi-square were used to compare demographic and clinical characteristics between groups at baseline and follow-up. Within-group changes for clinical measures were assessed using paired t-tests. A mixed full factorial General Linear Model (GLM) was used to assess the effect of group (hip OA versus control) and time (baseline and 12-month follow-up) on each dependent measure. A priori contrasts were used to assess differences from baseline to follow-up within each group. A further GLM was used to assess the effect of group on percentage changes from baseline to follow-up. Statistical analyses were performed using SPSS version 24.0 for Windows (SPSS Inc., Chicago, USA) with significance level set at *p* < 0.05. Unless otherwise specified, all data are presented as the mean and one standard deviation.

## Results

Participants in the hip OA and control groups were not statistically different with respect to sex, age, height and mass (Table 1). Participants with hip OA had a higher BMI at baseline than controls (*p* = 0.03). Five participants with hip OA (36%) reported bilateral symptoms concurrent with imaging findings. In these cases, data are reported for the more symptomatic limb. The average time to follow-up did not differ between the hip OA (54 ± 3 weeks) and the control (55 ± 2 weeks) groups (*p* = 0.48). Harris Hip Scores in the hip OA group were not siginificantly different between baseline (mean = 70.7; range = 42.9–91.3) and follow-up (mean = 72.6; range = 48.4–95.7). Participants in the hip OA group had Kellgren-Lawrence grades of 2 (36%) and 3 (64%) at baseline, and 2 (43%), 3 (43%) and 4 (14%) at follow-up. Joint space width did not significantly worsen over the follow-up period, within either group (Table 1).

**Table 1:** Demographic and clinical characteristics of the hip osteoarthritis (n=14) and control (n=15) groups at baseline and 12-month follow-up.

	Baseline^¢^		Follow-up	
	Hip OA	Control	Hip OA	Control
Age (yrs)	64.5 ± 5.4	59.3 ± 10.0	65.6 ± 8.7	60.4 ± 9.9
Male sex, n (%)	3 (21)	6 (40)	3 (21)	6 (40)
Height (cm)	164.5 ± 10.6	169.8 ± 8.7	164.5 ± 10.7	169.7 ± 8.7
Mass (kg)	73.2 ± 11.1	71.1 ± 10.1	72.7 ± 11.0	71.2 ± 10.0
Body mass index (kg/m^2^)	27.0 ± 2.6^*^	24.6 ± 2.8	26.8 ± 2.4	24.7 ± 3.2
Harris Hip Score (HHS)^†¢^	69.9 (42.9-91.3)	100 (96.8-100)	72.6 (48.4-95.7)	100 (97.9-100)
HHS pain^η^	20.0 (10.0-40.0)	44.0 (44.0-44.0)	30.0 (10.0-44.0)	44.0 (44.0-44.0)
HHS function^Ω^	41.5 (29.0 -45.0)	47.0 (44.0-47.0)	39.5 (30.0-47.0)	47.0 (45.0-47.0)
Joint space width (mm)	2.56 ± 0.90^*^	4.17 ± 0.62	2.54 ± 1.20^*^	4.27 ± 0.49
Kellgren-Lawrence grade^δ^	KL 2 = 5 KL 3 = 9	KL 0 = 11 KL 1 = 4	KL 2 = 6 KL 3 = 6 KL 4 = 2	KL 0 = 8 KL 1 = 7

Values are mean (standard deviation), with the exception of HHS values which are median (range); *p < 0.05 – hip osteoarthritis group significantly different to control group; ¢Most symptomatic hip for participants with bilateral hip osteoarthritis and randomly assigned hip for control participants; †HHS scale – 0 = extreme hip problems and 100 = no hip problems; ηHHS pain subscale – 0 = extreme hip related pain and 44 = no hip related pain; ΩHHS function subscale – 0 = extreme hip related dysfunction and 47 = no hip related dysfunction; δKellgren-Lawrence grading scale – 0 = no radiographic features of hip osteoarthritis and 4 = large osteophytes; OA – osteoarthritis

### Hip and knee muscle strength

**Fig. 2:**
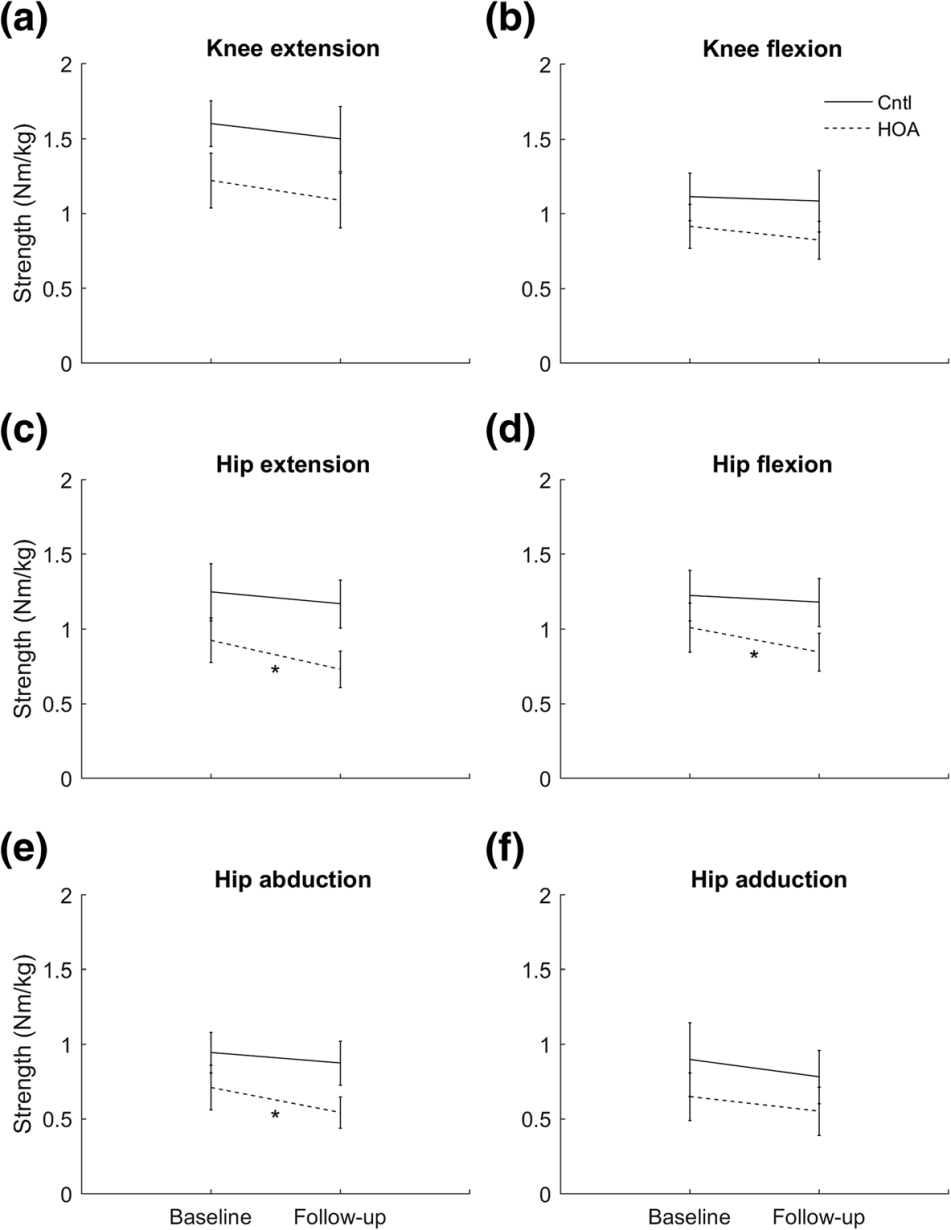
Muscle strength (mean ± one standard deviation) for hip osteoarthritis (*n* = 14) and control (*n* = 15) participants at baseline and 12-month follow-up; (**a**) knee extension, (**b**) knee flexion, (**c**) hip extension, (**d**) hip flexion, (**e**) hip abduction, and (**f**) hip adduction. Cntl – control group; HOA – hip osteoarthritis group; ^*^p < 0.05 – significant difference from baseline to follow-up in the hip osteoarthritis group

There was a significant main effect of group for knee extension (F = 9.510, *p* = 0.005, hip OA: 1.12 ± 0.33 Nm/kg, control: 1.55 ± 0.33 Nm/kg), hip extension (F = 11.360, *p* = 0.002, hip OA: 0.85 ± 0.28 Nm/kg, control: 1.21 ± 0.28 Nm/kg), hip flexion (F = 5.259, *p* = 0.031, hip OA: 0.93 ± 0.29 Nm/kg, control: 1.20 ± 0.29 Nm/kg), and hip abduction strength (F = 10.412, *p* = 0.003, hip OA: 0.63 ± 0.24 Nm/kg, control: 0.91 ± 0.24 Nm/kg). No significant group-by-time interactions were detected. However, a priori contrasts revealed a significant decline in strength from baseline to follow-up for the hip extensors (*p* = 0.047), hip flexors (*p* = 0.028) and hip abductors (*p* = 0.029) within the hip OA group only (Fig. 2).

### Lower extremity lean and fat mass, and thigh muscle size and quality

**Fig. 3:**
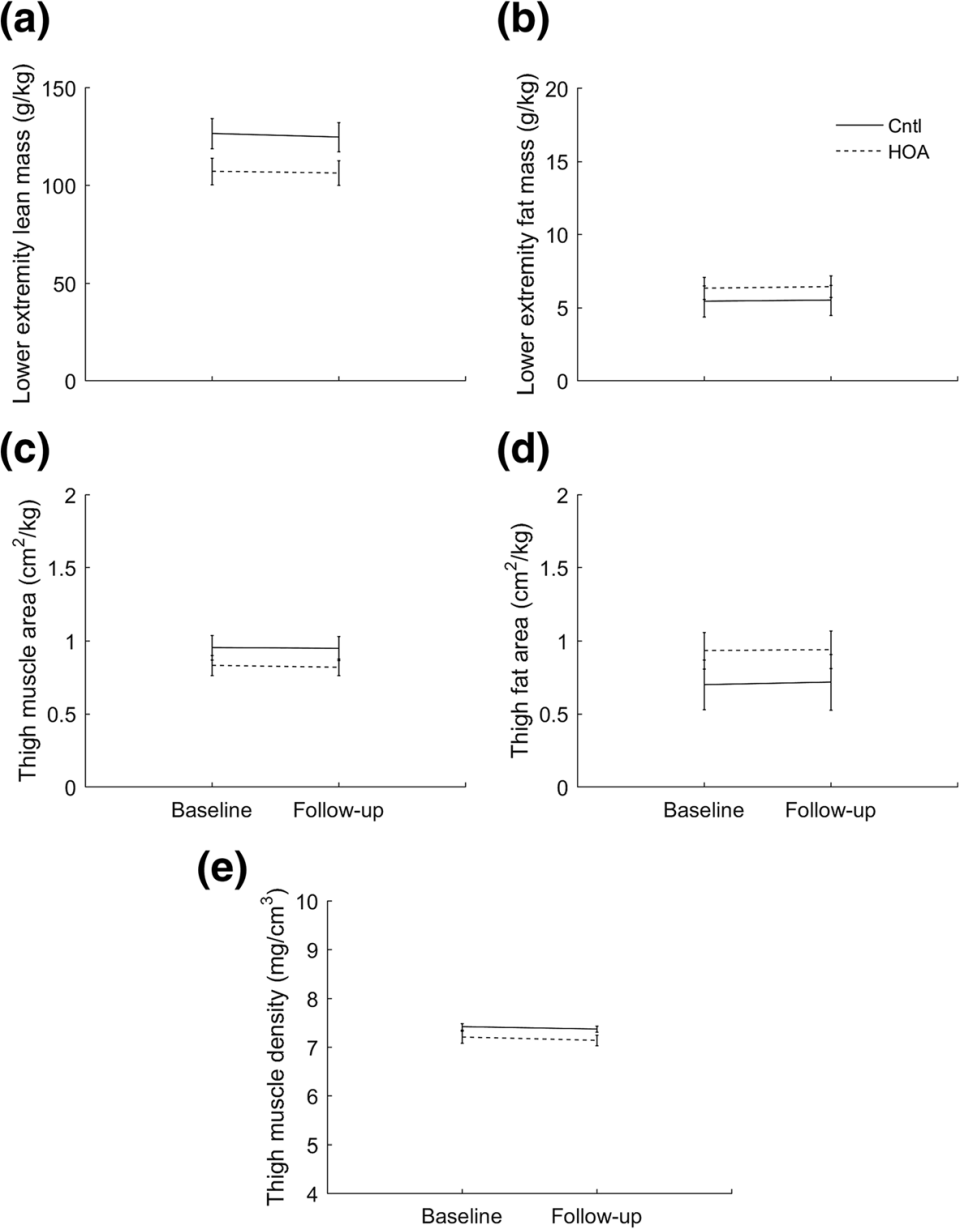
Muscle and fat measures (mean ± one standard deviation) for hip osteoarthritis (*n* = 14) and control (*n* = 15) participants at baseline and 12-month follow-up; (**a**) lower extremity lean mass, (**b**) lower extremity fat mass, (**c**) thigh muscle area, (**d**) thigh fat area, and (**e**) thigh muscle density. Cntl – control group; HOA – hip osteoarthritis group

There was a significant main effect of group for lower extremity lean mass (F = 13.220, *p* = 0.001, hip OA: 106.7 ± 13.5 g/kg, control: 125.7 ± 13.9 g/kg), thigh muscle area (F = 5.466, *p* = 0.027, hip OA: 0.825 ± 0.150 cm^2^/kg, control: 0.952 ± 0.150 cm^2^/kg), and thigh muscle density (F = 11.240, *p* = 0.002, hip OA: 7.17 ± 0.18 mg/cm^3^, control: 7.40 ± 0.18 mg/cm^3^) (Fig. 3). No significant group-by-time interactions were detected, and no within-group changes were detected over the follow-up period.

**Fig. 4:**
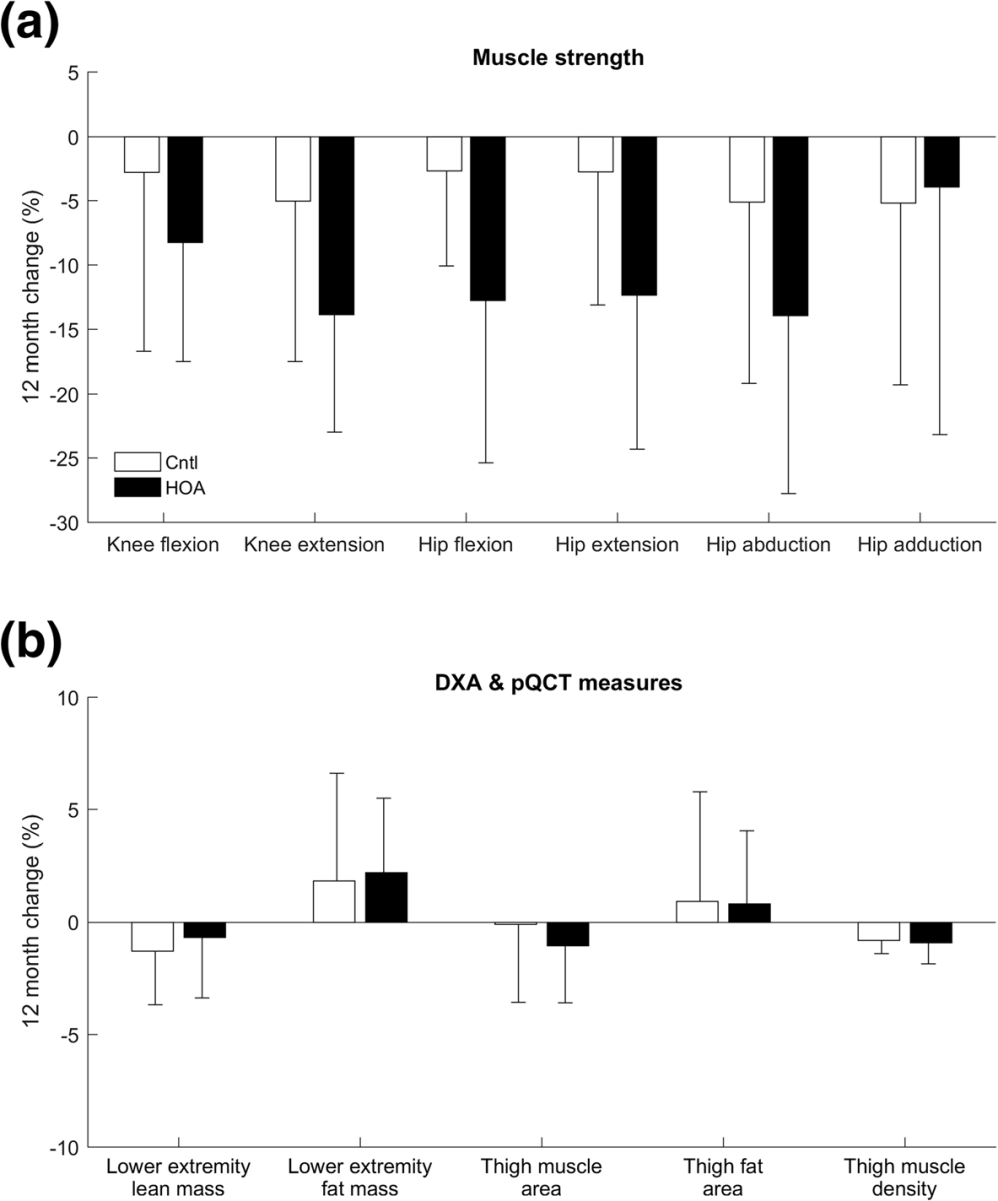
Within-group changes (follow-up – baseline) for (**a**) strength and (**b**) muscle and fat measures for hip osteoarthritis (*n* = 14) and control (*n* = 15) groups. Cntl – control group; DXA – dual-energy x-ray absorptiometry; HOA – hip osteoarthritis group; pQCT – peripheral quantitative computed tomography. No differences in 12-month changes were observed between-groups

No significant group differences in the percentage changes from baseline to follow-up were detected for any measure (Fig. 4).

## Discussion

This study prospectively evaluated changes in muscle strength, size and quality, and lower extremity lean and fat mass in individuals with mild-to-moderate hip OA relative to healthy controls without hip OA. Although deficits in hip and knee muscle strength, lower extremity lean mass, and thigh muscle size and density were evident in the hip OA compared to the control group, between-group differences were not significantly increased over the follow-up period. However, consistent with our hypothesis, within-group analysis revealed that muscle strength declined in the hip extensors, hip flexors and hip abductors over the follow-up period for the hip OA group, but not the control group. These findings suggest that lower limb muscle weakness is a defining feature of mild-to-moderate hip OA and reinforce the potential of targeted interventions to mitigate lower limb muscle weakness in this patient population.

### Hip and knee muscle strength

Strength deficits in the hip OA compared to the control group were detected for the knee extensors and hip extensors, flexors and abductors. Hip abductor muscle weakness has been identified in cohorts with pre-arthritic intra-articular hip pathology [31], and with early- [7] and late-stage OA [32, 33]. Findings from our data [22], and that of others [7, 32] suggest that the magnitude of strength deficits in individuals with hip OA compared to controls are substantial (~ 10–20%), even early in the disease course. These strength deficits may underpin the reduction in physical function reported in individuals with advanced hip OA [5]. An interesting observation was that strength deficits in our hip OA cohort were not limited to the muscle groups surrounding the affected joint. Studies of individuals with knee OA demonstrate significant weakness of the hip musculature compared to healthy controls [34] and that rehabilitation programs which included hip muscle strengthening decreased pain and improved function in these patients [35]. Further, greater strength of both hip and knee muscle groups has been associated with better function in individuals with hip OA from across the disease spectrum [36]. Taken together, our findings and those of others suggest that programs to prevent or slow the decline of both hip and knee muscle strength may also be relevant in the management of mild-to-moderate hip OA.

### Prospective changes in symptoms and joint structure

Self-reported symptoms (assessed via HHS) and measures of joint structure (assessed via KL grade and JSW measures) did not worsen significantly over the 12-month follow up period in the hip OA group. Only two participants progressed to more advanced hip OA (from KL grade 3 to 4) over the follow-up period, and no participants progressed to total hip replacement. A longer follow-up period may be required to identify factors that influence disease advancement, particularly in a cohort earlier in the OA trajectory, where progression is expected to be more gradual than in later stages [37]. Although structural progression was observed previously in a cohort of hip OA patients over a 12-month period, less than half of included patients worsened over this time frame [13]. Heterogeneity is common in OA populations [38], and may explain why we did not observe symptomatic or structural decline in the present study. The complex pathogenesis of hip OA means that individuals are likely to progress at different rates and via different mechanisms, consequently limiting the efficacy of uniform conservative treatments [39]. Longer-term prospective investigatons of larger cohorts are required to further understand factors that influence disease advancement across all affected tissues, and to appropriately define patient phenotypes and matched targeted interventions to slow disease progression.

### Prospective changes in hip and knee muscle strength

Contrary to our hypothesis, we observed no difference in 12-month change between groups for any measure of muscle strength. However, within-group analysis revealed a significant decline in hip extensor, hip flexor and hip abductor strength from baseline to follow-up in the hip OA group. No such declines in strength were identified within the control group. Strength declines over the follow-up period in the hip OA group were 2–3 times higher than in the control group, and 1.5–2 times the minimal detectable change. The combined effect of pre-existing muscle weakness at baseline and further decline over follow-up meant the hip OA group was on average, one-third weaker than the control group after 12-months. Strength deficits at follow-up were particularly apparent in the hip flexors and hip abductors (> 30%), muscle groups which make essential contributions to hip joint loading [16] and are critical for physical function.

### Lower extremity lean and fat mass, and thigh muscle size and quality

Participants in the hip OA group had significantly lower thigh muscle area, thigh muscle density and lower extremity lean mass compared to the control group on average by 15, 12 and 3% respectively. Overall, these differences were large relative to the measurement repeatability (coefficient of variation < 1%). Lower values for these parameters infer lesser muscle size and lower muscle quality, and likely underpin the observed hip muscle weakness in our hip OA cohort. However, unlike strength deficits, muscle size and quality did not deteriorate over the follow-up period. The increase in muscle weakness during the follow-up period in individuals with hip OA may instead be explained by muscle inhibition, perhaps as a direct consequence of pain or fear of pain.

We observed no differences in lower extremity fat mass or thigh fat area between groups. Obesity is increasingly recognized as an influential factor in knee OA progression through both local and systemic mechanisms [20], though any relationship with hip OA progression is less clear. Participants in the hip OA group had a higher BMI than control participants at baseline, but not at follow-up. However, measures of BMI [40] and thigh fat may not be representative of fat distribution within a person and so better measures of fat distribution may be warranted in future studies. Nevertheless, the substantial strength deficits and associated muscle atrophy observed in our cohort may suggest that muscle characteristics are a more appropriate marker of mild-to moderate hip OA than local fat, at least over the short-term.

### Strengths and limitations of the study

This is the first known investigation to assess longitudinal changes in muscle and fat characteristics in individuals with mild-moderate hip OA relative to controls. Eligibility for both groups were based on radiographic and symptomatic criteria, which minimized the risk of participant misclassification [41]. Findings were unchanged when the statistical model was adjusted for BMI at baseline (results not presented). This study had several limitations. First, although within-group declines in muscle strength were detected over the follow-up period, the study may not have been sufficiently powered to detect between-group changes or within-group changes in other outcome measures quantified using DXA and pQCT. There was however a tendency for lower extremity lean mass, thigh muscle area and density to decline more, and fat area and mass to increase more in the hip OA group relative to the control group. As no prior studies have prospectively evaluated changes in muscle strength in mild-to-moderate hip OA, no data were available to estimate sample size for the present study. The effect sizes from the present study may therefore serve as a guide for the design of larger prospective studies conducted over a longer follow-up period. Second, strength was assessed under isometric conditions, which may not reflect muscle function during dynamic conditions including activities of daily living. Participant pain was assessed with the HHS, but was not directly evaluated during strength testing which could have influenced our results. Third, measures from pQCT were taken from a single slice through the thigh, which may not be representative of changes in muscle and fat characteristics in other regions of the lower limb. Fourth, it is not clear whether between-group differences in muscle and fat characteristics observed at baseline preceeded the onset of hip OA or occured as a consequence of the disease. Subsequent prospective studies will be required to answer this question and to identify further mechanisms underlying disease progression. These studies should aim to include additional potentially relevant participant data like physical activity level and symptom duration. Last, observations from this study aim to inform hypotheses to be tested in larger studies, and as such we did not correct for multiple statistical comparisons.

## Conclusions

Findings from this exploratory study suggest that pre-existing deficits in hip muscle strength within the hip OA group were accentuated over the 12-month follow-up period, whereas observed between-group differences in muscle size and quality at baseline did not worsen over time. Interventions to prevent or slow decline in hip and knee muscle strength may be relevant in the management of mild-to-moderate hip OA.

## Additional file

Additional file 1: **Figure S1.** Maximal voluntary isometric strength testing position for hip flexors, extensors, abductors and adductors. (PDF 125 kb)

## Data Availability

The datasets used and/or analyzed during the current study are available from the corresponding author on reasonable request.

## Abbreviations

DXA: Dual-energy x-ray absorptiometry
GLM: General Linear Model
HHS: Harris hip score
JSW: Joint space width
KL: Kellgren-Lawrence
OA: Osteoarthritis
pQCT: Peripheral quantitative computed tomography
