# Supplementary figures and images for: A 12-month prospective exploratory study of muscle and fat characteristics in individuals with mild-to-moderate hip osteoarthritis
Source: BMC Musculoskelet Disord. 2019 Jun 14;20:283. doi: 10.1186/s12891-019-2668-z (PMC6570923; doi:10.1186/s12891-019-2668-z)

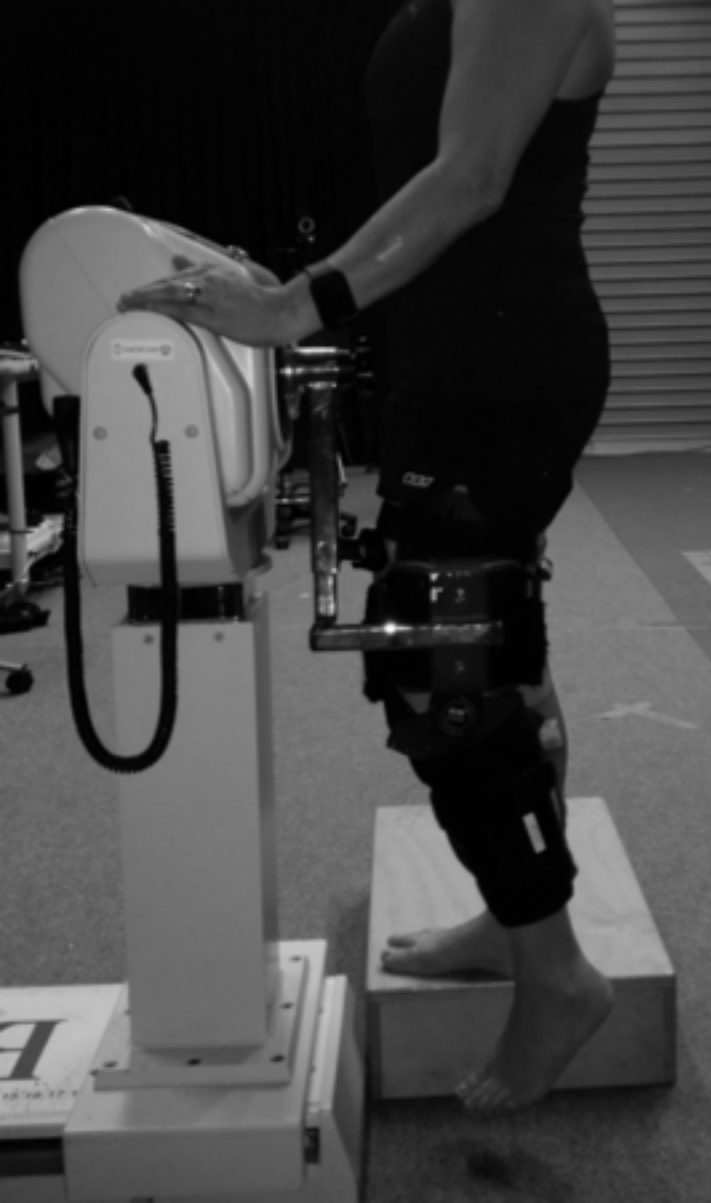

Supplement: Supplementary file 1 — Figure S1. Maximal voluntary isometric strength testing position for hip flexors, extensors, abductors and adductors. (PDF 125 kb) [file 12891_2019_2668_MOESM1_ESM.pdf]
